# Comparison of vermicompost characteristics produced from sewage sludge of wood and paper industry and household solid wastes

**DOI:** 10.1186/s40201-017-0269-z

**Published:** 2017-03-09

**Authors:** A.I. Amouei, Z. Yousefi, T. Khosravi

**Affiliations:** 1 Department of Environmental Health Engineering, Babol University of Medical Sciences, Babol, Iran; 2 Environmental Health Research Center (EHRC), Babol University of Medical Sciences, Babol, Iran; 3 Department of Environmental Health Engineering, School of Public Health, Mazandaran University of Medical Sciences, Sari, Iran

**Keywords:** Vermicompost, Earthworm, Eisenia fetida, Household solid waste, Biological and chemical sludges

## Abstract

**Background:**

The aim of this study was to determine the potential of produced compost from the sludge of wastewater treatment plant using earthworms and compare it with the vermicompost produced from household solid waste.

**Methods:**

In the current study, three treatments with the same conditions in terms of organic wastes type were prepared to produce vermicompost from household solid waste and sewage sludges using earthworms. The standard methods were used to determine the physical and chemical parameters in the different produced vermicomposts.

**Results:**

The mean of C/N in the household solid waste, raw biological and chemical sludges was 32, 22.5, and 26.5, respectively. These levels were 16.5, 14.5, and 15 in the vermicomposts. The mean of nitrogen and phosphorus percentages in the vermicompost of solid waste, biological and chemical sludges was 2.2%, 2.6%, 2.3% and 0.72%, 0.54%, and 0.56%, respectively. The mean percentages of organic matters in the initial substrates and vermicomposts of solid waste, biological and chemical sludges were 97.2%, 90%, 80.5% and 65.8%, 67.8% and 63% respectively. The concentrations of heavy metals decreased in all vermicomposts. The EC levels in solid waste, biological and chemical sludges were 1459, 1041, and 1487 μs/cm, respectively. These levels were 544, 385 and 635 μs/cm in the produced compost.

**Conclusion:**

Eisenia fetida can convert household solid waste, and biological and chemical sludges produced from wastewater treatment plant into a high-quality and acceptable compost.

## Background

Overuse of fertilizers and pesticides in agriculture as well as the discharge of sewage sludge and municipal waste have reduced soil quality and imposed great risks on human health and the environment [1, 2]. The installation and operation of sewage sludge stabilization reactors in small wastewater treatment plants with an average rate of less than 10 l per second are not cost-effective due to the high cost of sludge stabilization reactors and sludge dewatering and transfer systems. Many treatment plants are equipped with only one sludge dewatering and drainage system. After dewatering, the sludge is buried or transferred to landfills [3, 4]. Undesirable characteristics of sewage sludge such as undesirable odor, high concentrations of heavy metals and pathogens are associated with disposal costs and environmental pollution costs [5, 6]. Therefore, a simple and inexpensive technology for the treatment and use of sewage sludge are required to remove pathogens and stabilize the sludge [5, 7].

One of the cheap, effective, natural and environment-friendly methods is composting by earthworms or vermicomposting [8]. Aristotle, the famous Greek scholar, referred to earthworms as the earth’s intestines and considered their unique role in soil fertility [9]. Since 600 years ago, earthworms have been known as “environment’s managers” in the ecosystem [5]. Vermicomposting is defined as a method of decomposing organic matter by earthworms and microorganisms. By moving through materials and creating aerobic conditions, earthworms increase the aerobic activity of microorganisms [6]. The processes of breaking down, crushing, synthesis, and microbial and enzymatic enrichment occurs in the earthworms’ intestine, and therefore, the earthworms’ fecal matters are abundant with water-soluble nutrients that are easily absorbed by plants [5]. Due to the presence of humic compounds, macro and micro nutrient elements, amino acids and beneficial soil microorganisms, the vermicompost products can be used for fertilization and soil fertility and increasing plant growth [8]. The use of earthworms in the compost production process is a technology suitable for waste management which stabilizes organic substances [1], biodegradation and bioaugmentation of petroleum hydrocarbons [10] and greatly reduces the population of pathogens [7].

The most important earthworms used in the composting process are Udrilus eugeniae, Eisenia Andrei, Metaphire Californica, Eisenia fetida, and Perionyx excavates [6, 9]. According to numerous studies, Eisenia fetida (red Californian worms) is a bisexual worm that starts reproducing 3 months after its birth. Their reproduction continues throughout their whole life. Earthworms need moisture and aerobic conditions for reproduction. They mate during the night which takes 30–240 min where they produce a capsule. After 14–21 days, 4–20 worms are born from one capsule [6]. Earthworms can decompose municipal solid waste, household waste, garden waste, animal waste, and urban and industrial sewage sludge [11–13]. They are also used as biological adsorbents in detoxification and purification of soil from heavy metals, resistant hydrocarbons and some organic pesticides, increasing the population and strength of the enzymatic activity of beneficial soil microorganisms, improving soil quality and developing sustainable agriculture [10, 14, 15]. The use of vermicomposting in stabilizing sludge in the United States of America has resulted in 100% elimination of pathogens [1, 4].

Iran, with more than 60,000 t of household waste per day, in urban and rural areas, is subjected to substantial costs. The disposal of such volume of waste causes environmental problems and concerns.

Due to the presence of 60–70% of compostable materials in the household solid wastes in northern Iran [5, 12] and the abundance of earthworms, especially Eisenia Fetida, in Iran [12], the present study was investigated the potential of compost production from household solid wastes, biological and chemical sludges of wastewater treatment plants through Eisenia fetida.

## Methods

### Collecting household solid waste and sewage sludge

In this study, after mixing and random sampling and separating biodegradables, 120 kg of household wastes produced in Sari city were transferred to the laboratory in plastic bags. The necessary biological and chemical sludges taken from the settling basin of wastewater treatment plant of wood and paper industries in Sari were transferred to the laboratory after dewatering and thickening. Chemical treatment unit in which Alum, Calcium hydroxide and polyelectrolyte are used for wastewater treatment is located after biological treatment one.

### Collecting the Eisenia fetida earthworms

The Eisenia fetida earthworms were collected from the rural areas of Sari according to the identification keys and were kept in the laboratory for several days to become compatible with the environment.

### Experimental design

The household solid waste and the produced sludges were air-dried for 1 day by being distributed on plastic plates. The preliminary decomposition of dried raw samples was performed for 20 days by aerobic bacteria to pass the bacteria from the thermophilic phase and prevent earthworm mortality. After pre-decomposition and reduction of the temperature of the substrate material to the laboratory ambient temperature, 400 adult Eisenia fetida earthworms were placed on the top layer of the reactor substrate. A plastic 50 × 25 × 20-cm cube pot was used for preparing the reactor. A 5-cm layer of gravel in 10 mm diameter was used to maintain the upper layers and to reach the adequate oxygen. A 1- to 5-milimeter layer of sand with 2.5-cm height was used for proper drainage of excess water as well as reaching the needed oxygen. A 2.5-cm layer of manure mixed with the soil of the area with the ratio of 1 to 1 was used to provide an appropriate environment for growth and activities of the earthworms. Then, a 10-cm layer of the primary substrate, including household waste or sewage sludge, was placed on the lower layers. To regulate the carbon-to-nitrogen ratio suitable for earthworms’ activities, the waste and sludge was mixed with rice straw at a ratio of 3 to 1. To provide the adequate Carbon to Nitrogen ratio (C/N =30) in initial substrates, 850 g of dewatered sludge were mixed to 150 g of rice straw. All treatments were kept in the laboratory at the temperature of 25 ° C. The moisture level in the samples in the composting process was set to 60–80% using a sprinkler. The composting process lasted 70 days. One replication was considered for all samples and treatments.

### Chemical analysis

In order to accurately measure the chemical parameters affecting composting, the contents of each reactor were first mixed. Then, 50 g of the initial substrate and the produced compost were air-dried and milled. Standard methods were used for the chemical analysis of each parameter [16]. Temperature was daily measured by a bar thermometer. The moisture and ash contents were measured by using a gravimetric method at 105 °C within 24 h and 550 °C for 4 h, respectively. The pH and electrical conductivity were measured using a digital pH meter and an electrical conductivity meter, respectively. The organic carbon, nitrogen and phosphorus levels in the samples were measured using the Walkley-Black, Micro kjeldahl and Colorimetric methods, respectively [16]. In addition, the concentration of heavy metals such as lead, cadmium, nickel, chromium, and iron was measured through atomic absorption spectrometry [15, 17]. SPSS software version 19 was used for data analysis.

## Results

During the study, the ambient temperature fluctuated between 18 and 20 °C. The initial range of pH was from 6.5 to 7 in the substrate of household waste and sludge. The pH was 7.7–8.2 in the vermicompost. Table 1 shows the chemical parameters of interest in the initial substrate.

**Table 1:** The important physical and chemical parameters in the initial substrate of waste and different sludges

Parameter	^a^HSW	^b^BS	^c^CS
- Humidity (%)	71.8 ± 3.4	48.2 ± 1.4	53.8 ± 5
- Ash (%)	2.8 ± 0.8	9.4 ± 1.6	19.5 ± 1.5
- Organic Matter (%)	97.2 ± 1.5	90 ± 1.2	80.5 ± 3
- Total Carbon (%)	54 ± 0.8	50.3 ± 0.8	44.5 ± 1
- PH	6.5 ± 0.6	7.3 ± 0.5	6.7 ± 0.8
- EC (μs/cm)	1459 ± 3	1041 ± 15	1485 ± 6
- N (%)	1.7 ± 0.2	2.2 ± 0.4	1.5 ± 0.1
- P (%)	0.54 ± 0.1	0.3 ± 0.2	0.4 ± 0.1
- Cr^2+^ (mg/kg)	6.4 ± 0.6	9.5 ± 0.4	27 ± 2
- Pb^2+^ (mg/kg)	0.8 ± 0.4	13.4 ± 3	16 ± 7
- Cd^2+^ (mg/kg)	0.2 ± 0.1	0.6 ± 0.2	1.7 ± 0.4
- Ni^2+^ (mg/kg)	7.4 ± 0.3	19.8 ± 1.2	16.8 ± 7
- Fe^2+^ (mg/kg)	7561 ± 16	3927 ± 8	6098 ± 35
- C/N	31.8 ± 5	22.5 ± 3	26.5 ± 8

^a^Household Solid Wastes

^b^Biological sludge

^c^Chemical sludge

In Table 2, the physical and chemical parameters in the vermicompost produced of wood and paper wastewater sludge and household solid w astes were presented.

**Table 2:** The important physical and chemical parameters in the vermicompost produced after 8 weeks

Parameter	HSW	BS	CS
- Humidity (%)	28.3 ± 1.8	32.2 ± 3.4	21.2 ± 3
- Ash (%)	34.1 ± 1.4	32.2 ± 1.5	39.5 ± 1.8
- Organic Matter (%)	65.8 ± 3.5	67.8 ± 3.2	63 ± 3
- Total Carbon (%)	36.6 ± 1.8	37.6 ± 2.2	33.5 ± 2.5
- pH	8.2 ± 0.4	8 ± 0.5	7.7 ± 1.2
- EC (μs/cm)	545 ± 5	358 ± 4	636 ± 12
- N (%)	2.2 ± 0.4	2.6 ± 0.5	2.2 ± 0.3
- P (%)	0.72 ± 0.3	0.5 ± 0.3	0.6 ± 0.2
- Cr^2+^ (mg/kg)	0	0	0
- Pb^2+^ (mg/kg)	0	0	2 ± 0.3
- Cd^2+^ (mg/kg)	0.05 ± 0.03	0.12 ± 0.1	0.8 ± 0.5
- Ni^2+^ (mg/kg)	0	0	0
- Fe^2+^ (mg/kg)	3915 ± 10	1506 ± 6	4412 ± 15
- C/N	16.5 ± 3	14.5 ± 2	15 ± 6

**Fig. 1:**
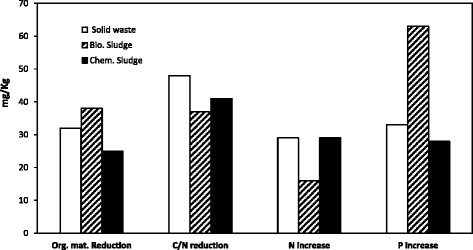
Comparison of various parameters in the vermicompost produced after 8 weeks

In Fig. 1, some characteristics of vermicompost that were produced from wastewater sludges and household solid wastes were presented.

Table 3 shows the concentration of some heavy metals in the soil samples.

**Table 3:** Heavy metals concentration of the soil samples (as mg/kg)

Element	1Soil	Soil2	Mean
Cr^2+^	40.2	40.2	40.2
Pb^2+^	18.1	18.3	18.2
Cd^2+^	1.6	1.4	1.5
Ni^2+^	39.8	39.6	39.7
Fe^2+^	19294	19286	19292

In Table 4, the content of the heavy metals in the different generated vermicomposts and soil samples were presented.

**Table 4:** Heavy metals concentration of soil and compost samples (as mg/kg)

Element	^a^Mixed	Mixed^b^	Mixed^c^
Cr^2+^	35.9	31.6	38.1
Pb^2+^	18.1	17.1	20.1
Cd^2+^	1.5	1.55	2.2
Ni^2+^	33.2	27.7	31.8
Fe^2+^	15376	17786	23689

^a^Mixed (Soil + HSW compost)

^b^Mixed (Soil + BS compost)

^c^Mixed (Soil + CS compost)

The results of experiments on vermicompost produced, compared with the amounts of the World Health Organization are presented in Tables 5 and 6.

**Table 5:** Comparison the chemical composition of different types of the produced composts with the WHO’s results [24]

Parameter	HSWV^a^	BSV^b^	CSV^c^	WHO^d^
Organic Mater (%)	65.9	68	61	10–30
Ash (%)	34.2	32.1	39.8	30–70
Total Nitrogen (%)	2.2	2.6	2.3	0.1–1.8
Total Phosphorous (%)	0.7	0.5	0.6	0.1–1.7
pH	8.2	8.2	7.8	6–9

^a^Household solid wastes vermicompost

^b^Biological sludge vermicompost

^c^Chemical sludge vermicompost

^d^World Health Organization

**Table 6:** Comparison the concentration of heavy metals in the different types of the produced composts with the WHO’s results

Elements	HSV	BSV	CSV	WHO
Iron (mg/kg)	3915	1506	4412	8000–15000
Lead (mg/kg)	0	0	1.9	200–400
Chromium (mg/kg)	0	0	0	----------
Cadmium (mg/kg)	0.05	0.1	0.8	15–40

## Discussion

According to the results and comparing Tables 1, 2, 3 and 4, it can be concluded that during the process of vermicomposting, significant physical and chemical changes had occurred in the raw materials of solid waste and all sludge due to the biodegradation of organic matter and the interactions between earthworms and microorganisms [14]. This phenomenon improves the properties of the final compost regarding the impact on the soil fertility. One of the most important physicochemical changes was the increased availability of nutrients to plants which occurs due to the activity of earthworms and microorganisms and the mineralization of organic materials [18, 19].

### Variations in pH

Earthworms and microorganisms are able to change the soil pH [20]. pH increased in all samples during the composting period. Intense microbial activity and decomposition of organic matter in the first weeks resulted in the formation of ammonium and increased pH of the compost [13, 20]. In the final stage of the experiment, the activity of microorganisms affecting the decomposition increased during the process of aerobic metabolism and in the presence of sufficient moisture which results in the production of alkaline products [12, 14]. At the final stage of the experiment, the pH dropped which could be attributed to the production of carbon dioxide due to the metabolic activities of earthworms and microorganisms.

### Variations in electrical conductivity (EC)

Results showed that electrical conductivity decreased due to the activity of earthworms and the decomposition of organic matter. This can be attributed to the biological accumulation of some minerals in the earthworms’ bodies, and consequently, the reduced amount of minerals in soil. Kharrazi et al. reported an increased electrical conductivity after the experiment period [21]. In their study, due to the activity of earthworms, decomposition of organic matter, and mineralization of compounds, their solubility and mobility were increased leading to increased electrical conductivity of the substrate material during the vermicomposting process.

### Variations in total organic carbon

Over time, organic matter decreased in the substrate in all samples. Reduction of the total organic carbon is due to the mineralization and decomposition of organic matter by earthworms in the substrate material and the loss of carbon compounds in Co_2_. The number of earthworms in substrate decreased due to the reduction of C/N ratio during the process which increased the oxidation of organic matter and reduced the organic carbon.

### Variations in nitrogen (N)

The nitrogen level increased over time, which was mainly due to the reduction of dry weight of organic matter in the substrate due to the decomposition by earthworms. Another reason for this increase was the presence and activity of earthworms in the substrate and secretion of enzymes by them.

### Variations in phosphorus (P)

Total phosphorus increased over time. This indicates the mineralization and mobilization of phosphorus due to the presence of microorganisms and enzymes in the intestines of earthworms [22], the mineralization of the organic matter in the substrate, and the reduction of the dry weight of the substrate. Kharrazi et al. reported an increase in nitrogen and phosphorus level and a decrease in C/N ratio during the experiment [21]. Parveresh [14] reported a decrease in phosphorus level after the experiment, which was attributed to the adsorption of inorganic phosphorus released from the earthworms’ tissues. Our results are inconsistent with the results of Parveresh and it may be due to the difference in the duration of the process, the quality of materials consumed by the earthworms, and the experiment conditions.

### Variations in the ratio of carbon to nitrogen (C/N)

Carbon to nitrogen (C/N) ratio represents the decomposition of organic compounds and the stability obtained during the composting process. C/N ratio of the substrate decreased in all the samples over time. This can be attributed to the decomposition of organic matter and the release of a portion of organic carbon as carbon dioxide and the mineralization of nitrogen due to the microbiological decomposition processes and the production of enzymes, mucous and nitrogen compounds [23]. Parvaresh reported a decrease in C/N ratio after the experiment period [14] which was consistent with the present results. The results in Table 5 showed that the amounts of ash, phosphorus, and pH are in the standard range recommended by WHO. The amount of organic matter was higher in this study than the recommended levels by WHO [24], which can be attributed to the use of solid wastes and sludges containing high organic matter. Vigueros et al. [4] reported the organic matter level as 60%, the total nitrogen as 2.5%, and the phosphorus level as 0.96%. The organic matter, nitrogen, and phosphorus levels in the present study were consistent with it results. Nas et al. studied the chemical characteristics of the vermicompost produced from different mixtures of animal manure, garden waste, and kitchen waste [1]. They reported an increase in phosphorus and nitrogen levels at the end of the experiment period. The amount of organic matter, C/N ratio, electrical conductivity, and pH was reduced in the final vermicompost due to the presence and activity of the earthworms.

### Concentration of heavy metals

Table 6 represents that the measured heavy metals in the vermicompost produced from waste and sewage sludge are in the standard range recommended by WHO. Results showed that the heavy metal concentrations decreased over time, indicating that earthworms were able to biologically accumulate heavy metals in their tissues. Heavy metals are accumulated in the earthworms’ tissues through two different methods: through direct skin contact with the nutrients dissolved in the soil, or by digestion of certain elements in soil and subsequent absorption through the intestines [11, 15]. The present results are consistent with the results of Shahmansouri et al.’ study [17]. Our results are also consistent with those of Naouni et al. [15], and Alidadi et al. [25]. Results of the current study indicate that applying Eisenia fetida earthworms in the full-scale works will be economic, because Yousefi et al. [26] showed in their study, Eisenia fetida earthworms were indigene in the area.

### Comparison the vermicompost quality in the household solid waste with sewage sludge

The percentage of humidity and organic matter was higher in the vermicompost produced from biological sludge than that of the compost produced from household solid waste and chemical sludge. Microorganisms are involved in the biological treatment process and decompose the organic matter in the sewage [27]. Therefore, the resulting biological sludge contains microorganisms whose bodies are considered organic compounds. Ash, EC, and heavy metals were at the highest level in the vermicompost produced from chemical sludge, whereas the amount of organic matter and the pH were at the lowest level in such vermicomposts. This can be due to the use of coagulants, such as lime or alum, which leads to the production of a sludge with increased ash, increased mortality of microorganisms due to reduced organic matter and reduced pH, and increased precipitation of heavy metals in the sludge produced from treatment plants. The mean of C/N ratio in the primary substrate of household solid waste, biological and chemical sludge was 32, 22.5 and 26.5, respectively. The mean of C/N ratio in the vermicompost produced from household solid waste, biological and chemical sludge was 16.5, 14.5, and 15, respectively. This indicates that the proportion of raw materials was optimal, and the best C/N ratio was present in the produced vermicomposts (The C/N ratios in the final product should range from 10 to 20, with an average of 15).

## Conclusion

Generally, it can be concluded that the vermicompost quality is the most important criterion in the recycling of organic waste and in agriculture as fertilizers. Eisenia fetida earthworms are able to convert municipal solid waste and biological and chemical sludges into appropriate quality compost therefore they contribute to the environmental protection. Average C/N in vermicomposting of sludges was higher than in solid wastes. In addition, nitrogen and phosphor of sludge compost increased, but heavy metals and EC decreased. Results indicated that *Eisenia fetida* can efficiently in addition to municipal solid waste, biological and chemical sludges convert into accepted fertilizer.
